# Recent incidence and surgery trends for prostate cancer: Towards an attenuation of overdiagnosis and overtreatment?

**DOI:** 10.1371/journal.pone.0210434

**Published:** 2019-02-04

**Authors:** Sabrina Jegerlehner, Arnaud Chiolero, Drahomir Aujesky, Nicolas Rodondi, Simon Germann, Isabelle Konzelmann, Jean-Luc Bulliard, on behalf of the NICER Working Group

**Affiliations:** 1 Division of General Internal Medicine, Inselspital, Bern University Hospital, University of Bern, Bern, Switzerland; 2 Division of Chronic Diseases, IUMSP, Lausanne University Hospital (CHUV), Lausanne, Switzerland; 3 Institute of Primary Health Care (BIHAM), University of Bern, Bern, Switzerland; 4 Observatoire valaisan de la santé (OVS), Sion, Switzerland; 5 Department of Epidemiology, McGill University, Montreal, Canada; Centro per lo Studio e la Prevenzione Oncologica, ITALY

## Abstract

**Background:**

Screening for prostate cancer is frequent in high-income countries, including Switzerland. Notably due to overdiagnosis and overtreatment, various organisations have recently recommended against routine screening, potentially having an impact on incidence, mortality, and surgery rates. Our aim was therefore to examine whether secular trends in the incidence and mortality of prostate cancer, and in prostatectomy rates, have recently changed in Switzerland.

**Methods:**

We conducted a population-based trend study in Switzerland from 1998 to 2012. Cases of invasive prostate cancer, deaths from prostate cancer, and prostatectomies were analysed. We calculated changes in age-standardised prostate cancer incidence rates, stratified by tumor stage (early, advanced), prostate cancer-specific mortality, and prostatectomy rates.

**Results:**

The age-standardised incidence rate of prostate cancer increased greatly in men aged 50–69 years (absolute mean annual change +4.6/100,000, 95% CI: +2.9 to +6.2) between 1998 and 2002, and stabilised afterwards. In men aged ≥ 70 years, the incidence decreased slightly between 1998 and 2002, and more substantially since 2003. The incidence of early tumor stages increased between 1998 and 2002 only in men aged 50–69 years, and then stabilised, while the incidence of advanced stages remained stable across both age strata. The rate of prostatectomy increased markedly until 2002, more so in the 50 to 69 age range than among men aged ≥ 70 years; it leveled off after 2002 in both age strata. Trends in surgery were driven by radical prostatectomy. Since 1998, the annual age-standardised mortality rate of prostate cancer slightly declined in men aged 50–69 years (absolute mean annual change -0.1/100,000, 95% CI: -0.2 to -0.1) and ≥ 70 years (absolute mean annual change -0.5/100,000, 95% CI: -0.7 to -0.3).

**Conclusions:**

The increases in the incidence of early stage prostate cancer and prostatectomy observed in Switzerland among men younger than 70 years have concomitantly leveled off around 2002/2003. Given the decreasing mortality, these trends may reflect recent changes in screening and clinical workup practices, with a possible attenuation of overdiagnosis and overtreatment.

## Introduction

Prostate cancer is common and a frequent cause of cancer death in the US, and many western European countries [1]. For instance in Switzerland, it accounts for 30% of all male new cancer cases and 15% of all male cancer deaths [2]. By the early 1990s, several medical societies issued guidelines for routine screening with prostate-specific antigen (PSA) testing with or without digital rectal examination, initially without evidence from randomised trials supporting screening effectiveness [3]. The two largest trials, the European Randomised Study of Screening for Prostate Cancer (ERSPC) and the Prostate, Lung, Colorectal, and Ovarian (PLCO) Cancer Screening Trial indicate that screening could reduce prostate cancer mortality, but with substantial harms notably due to overdiagnosis and overtreatment [4, 5]. Several organisations, including the US Preventive Services Task Force (USPSTF) in 2012 and the Swiss Medical Board in 2011 therefore recommended against routine PSA-based screening [6, 7]. In 2018, results from the Cluster Randomized Trial of PSA Testing for Prostate Cancer (CAP) in UK showed that even a low-intensity strategy aiming to reduce overdiagnosis leads to an increased detection of low-risk prostate cancer cases, without decrease in prostate cancer mortality [8]. The same year the USPSTF has partly upgraded its recommendation to support individualized decision making for prostate cancer screening (C recommendation) in men aged 55 to 69 only [9].

Changes in screening practice has effects on prostate cancer incidence. Hence, following the 2012 USPSTF recommendation against screening, prostate cancer incidence has decreased in the US, predominantly for early-stage and low-grade cancer, without substantial concurrent changes in mortality [10]. If screening causes overdiagnosis and overtreatment [11–15], then less screening -and associated workup practice- should lead to reductions in both incidence and surgery rates for prostate cancer. To date, however, whether the decrease in incidence is paralleled with a decrease in the rate of prostatectomy has not been shown. To our knowledge, no previous study has analysed concomitantly secular trends in incidence, mortality, and surgery of prostate cancer in a whole country.

Our aim was therefore to assess recent secular trends in incidence, mortality, and surgery for prostate cancer using exhaustive and high quality data on the whole population of Switzerland.

## Methods

### Study design

We conducted population-based temporal trend analyses of incidence and mortality rates of prostate cancer, and of prostatectomy rates in Switzerland between 1998 and 2012.

### Data sources and case definition

#### Registry data for cancer cases and mortality

In Switzerland, cancer registration is organised regionally with a high level of completeness [16]. Data on all new cancer cases are collected, documented, and recorded by population-based regional cancer registries. The National Institute for Cancer Epidemiology and Registration (NICER, www.nicer.org) compiles and aggregates this data. Quality control procedures are based on the guidelines from the European Network of Cancer Registries [17]. In 2012, NICER data covered 68% of the Swiss population.

For our analyses, we used all prostate cancer cases (International Classification of Diseases for Oncology, 3^rd^ edition (ICD-O-3) [18]: C61) recorded in Swiss cancer registries between 1998 and 2012. Tumor stage (early, advanced) was defined according to recent oncology guidelines using the pathological TNM classification (www.uicc.org/resources/tnm). Stage I (T1-2a) and stage II (IIA = T2b, IIB = T2c) tumors were defined as early (localised) cancers, whereas stage III (T3a-b) and stage IV (T4N0M0, all N1, all M1) tumors were defined as advanced cancers. Trends by stage were limited to Swiss regions for which staging was documented in over 90% of cases each year (Fribourg, Geneva and Valais) to allow reliable analyses. Grading was available but could not be used due to substantial Gleason score reclassification over time, a known artefactual shift in grading of prostate cancer by pathologists [19].

Analyses were stratified across three age groups according to usual screening activity: under 50 years (screening not recommended), 50–69 years (screening recommended or not depending on medical society), and 70 years and above (screening usually not recommended).

#### Hospital data for prostate cancer surgery

Data on prostate cancer surgery were collected from all Swiss inpatient cases using the Federal Statistical Office Hospital Medical Statistics. Surgical procedures are registered by a year-specific Swiss Classification of Surgical Interventions (CHOP) code [20]. Codes are determined by physicians and checked by trained medico-administrative staff to ensure their accuracy and completeness. In 2012, 99% of all hospitals in Switzerland were included with a case coverage of 98% of all admissions.

To analyse trends in surgery between 1998 and 2012, we used year-specific diagnostic ICD-O-3 and CHOP codes. We identified individuals with a diagnosis of prostate cancer (ICD-O-3 code: C61) who had prostatectomy during the same year. To distinguish prostatectomies specifically intended for cancer from prostatectomies for other indications resulting in cancer diagnosis after histological workup, we considered separately “radical prostatectomy” (CHOP code: 60.5; radical prostatectomy) and “other prostatectomy” (CHOP code: 60.2, 60.3, 60.4; transurethral resection of the prostate, suprapubic prostatectomy, retropubic prostatectomy).

#### Statistical analyses

Rates were age-standardised to the most commonly applied 1976 European standard population using mid-year population estimates. We computed annual prostate cancer incidence rates by tumor stage (early, advanced) and age group (< 50 years, 50–69 years, ≥ 70 years). We further computed annual prostate cancer specific mortality and prostatectomy rates by age group, and surgical procedure (“radical prostatectomy” versus “other prostatectomy”). We fitted a linear regression model to estimate the annual mean absolute and relative changes in the standardised rates, with calendar year as predictor variable. Joinpoint statistical software (version 4.3; Surveillance Research Program, National Cancer Institute, Bethesda, MD) was used to identify and estimate the parameters of the linear model, to provide their standard errors allowing for heteroscedasticity management, and to test for statistical significance of trends. Cut-points obtained for incidence, mortality, and prostatectomy trends were used for the sub-analysis stratified by age group or tumor stage. To assess the robustness of the defined age groups and of our *a priori* strict inclusion criterion for the degree of stage completeness, we performed sensitivity analyses excluding men 70–74 years and including regions with at least 60% of stage information, respectively. Statistical analyses were performed with STATA (version 14) and R (version 3.3.1).

#### Ethics

Only de-identified and publicly available aggregated data were used. There was no threat to patient confidentiality. According to the Swiss Human Research Act (Humanforschungsgesetz), no ethical approval or trial registration is needed for such analyses. Our study protocol was agreed upon by NICER. The SFSO allowed the analyses and publication of predefined hospital data (contract 150 556).

## Results

**Fig 1:**
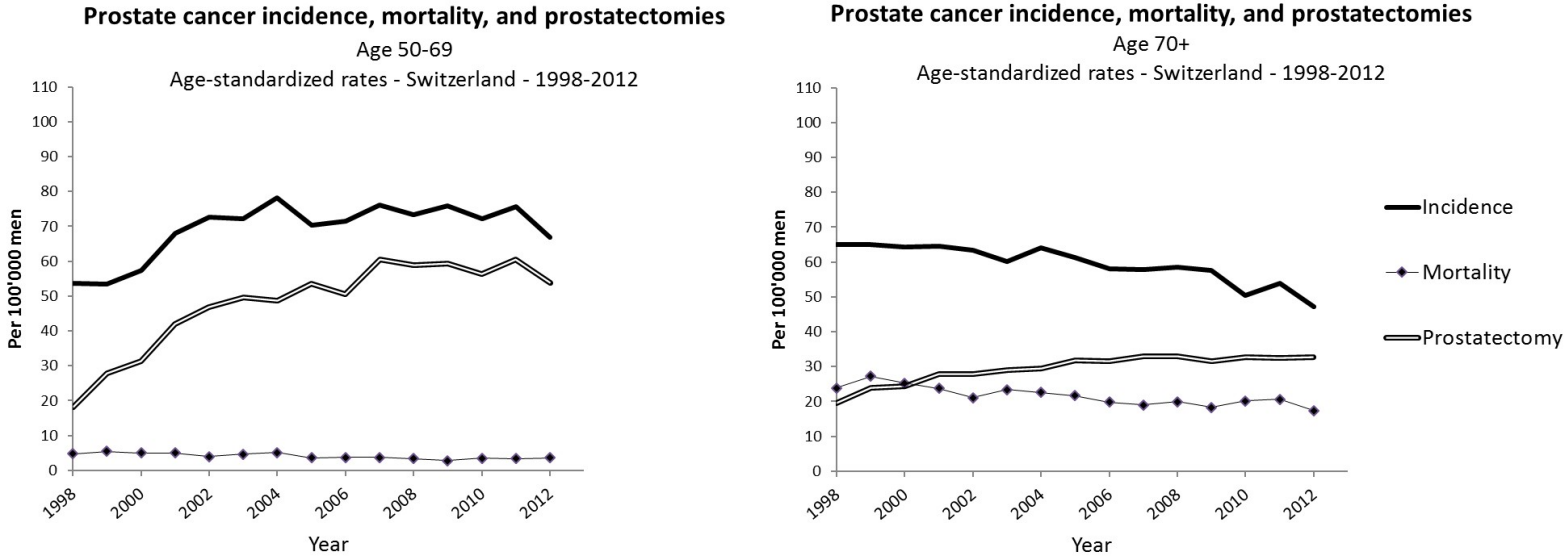
Age-standardised (European population) incidence, mortality, and prostatectomy rates for prostate cancer by age group (A: 50–69 years, B: ≥ 70 years) in Switzerland, 1998–2012. Data source: National Institute of Cancer Epidemiology and Registration (NICER) and Federal Statistical Office (FSO) Hospital Medical Statistics.

Between 1998 and 2012, 51,986 new cases of prostate cancer were registered. The number of cases and deaths, and the age-adjusted incidence and mortality rates by year and age group are reported in S1 Table. Between 1998 and 2003, the incidence of prostate cancer increased from 53.7/100,000 to 72.2/100,000 in men aged 50–69 years, corresponding to a 7% annual increase (95%CI: +5% to +10%) and an absolute mean annual change of +4.6/100,000 (95%CI: +2.9 to +6.2) whereas the incidence was overall stable from 2003 to 2012 (Table 1, Fig 1 **panel A**).

**Table 1:** Trends in the incidence and mortality of prostate cancer, and in the incidence of prostatectomy in Switzerland by age group, 1998–2012. All rates are directly standardised to the European population (per 100,000 inhabitants).

	50–69 years old				70+ years old			
	Absolute annual mean change in rate [per 100,000]	95% CI	Relative change per year [%]	95% CI	Absolute annual mean change in rate [per 100,000]	95% CI	Relative change per year [%]	95% CI
**Incidence**								
*1998–2003*	+4.6	[2.9;6.2]	+7%	[5;10]	-0.8	[-1.4;-0.2]	-1%	[-2;0]
*2003–2012*	-0.3	[-1.1;0.5]	0%	[-1;1]	-1.5	[-2.2;-0.9]	-2%	[-3;-1]
**Incidence by stage**^†^								
Early								
*1998–2003*	+4.2	[2.2;6.2]	+11%	[6;16]	+0.7	[-2.1;3.5]	+3%	[-8;14]
*2003–2012*	+0.6	[0.8;2.0]	+1%	[-1;4]	+0.1	[-0.4;0.6]	0%	[-1;2]
Advanced								
*1998–2003*	-0.4	[-2.5;1.6]	-2%	[-9;6]	+1.1	[-0.5;2.6]	+6%	[-3;16]
*2003–2012*	0.0	[-0.6;0.6]	0%	[-3;3]	-0.2	[-0.6;0.2]	-1%	[-4;1]
Unknown								
*1998–2003*	0.0	[-0.3;0.4]	+1%	[-15;18]	-0.4	[-1.0;0.2]	-6%	[-14;3]
*2003–2012*	+0.4	[0.2;0.6]	+30%	[13;48]	+0.2	[-0.2;0.5]	+3%	[-3;9]
Mortality*	-0.1	[-0.2;-0.1]	-3%	[-4;-1]	-0.5	[-0.7;-0.3]	-2%	[-3;-1]
**Prostatec-tomy rate**^‡^								
All								
*1998–2002*	+7.6	[3.6;11.5]	+40%	[20;62]	+2.1	[0.9;3.3]	+10%	[5;16]
*2002–2012*	+1.1	[0.2;2.0]	+2%	[0;4]	+0.4	[0.2;0.6]	+1%	[1;2]
Radical								
*1998–2002*	+6.9	[5.5;8.2]	+53%	[43;64]	+1.3	[0.6;2.0]	+54%	[23;84]
*2002–2012*	+0.8	[0.0;1.6]	+2%	[0;4]	+0.5	[0.4;0.6]	+7%	[5;9]
Other								
*1998–2002*	+0.4	[-0.5;1.2]	+7%	[-8;21]	+0.9	[0.0;1.8]	+5%	[0;10]
*2002–2012*	+0.3	[0.1;0.4]	+4%	[2;6]	0.0	[-0.2;0.1]	0%	[-1;0]

Data sources:

*National Institute of Cancer Epidemiology and Registration (NICER);

^†^NICER limited to 3 regions (Fribourg, Geneva, Valais);

^‡^Federal Statistical Office (FSO) Hospital Medical Statistics.

In men aged 70 years and above, the incidence of prostate cancer decreased from 65.1/100,000 to 47.2/100,000 between 1998 and 2012, corresponding to a 1% annual decrease (95%CI: -2% to 0%) between 1998 and 2003, and a 2% annual decrease (95%CI: -3% to -1%) between 2003 and 2012 (Table 1, Fig 1 **panel B**).

**Fig 2:**
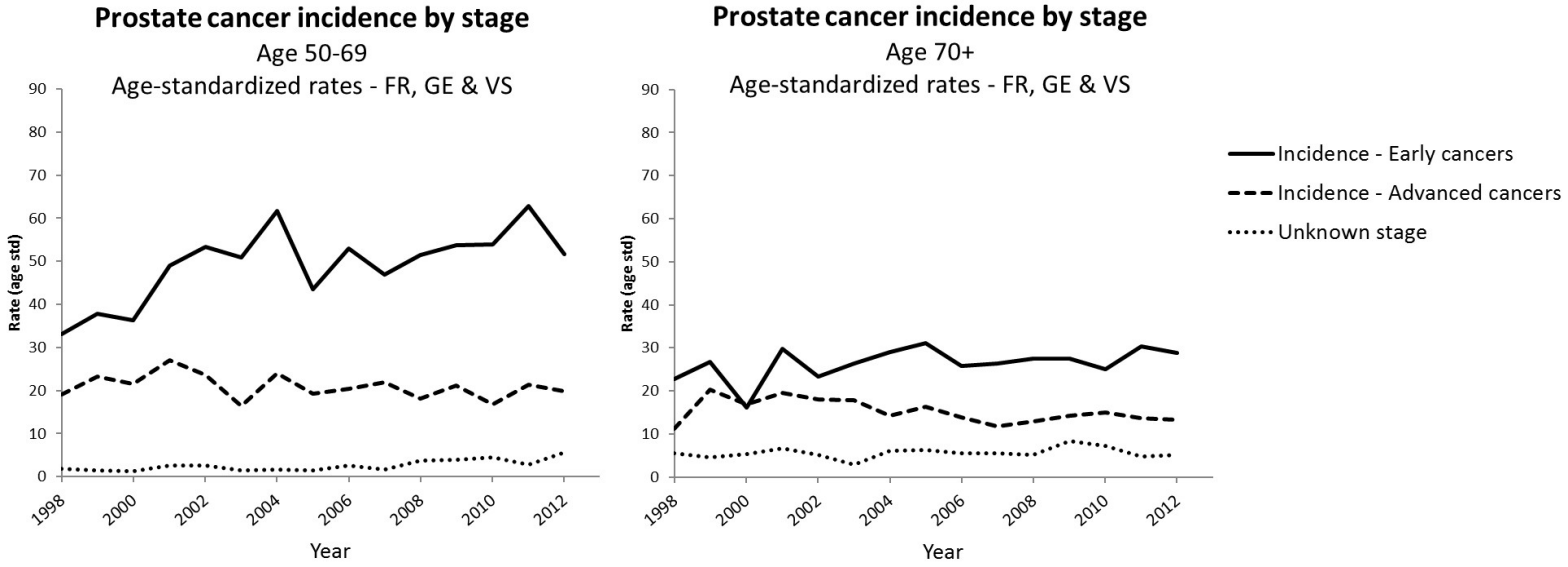
Age-standardised (European population) incidence rates of prostate cancer by stage and age group (A: 50–69 years, B: ≥ 70 years) in Switzerland, 1998–2012. Data source: National Institute of Cancer Epidemiology and Registration (NICER), limited to 3 regions (Fribourg (FR), Geneva (GE), Valais (VS)).

The incidence of early and advanced prostate cancer by year and age group is reported in S2 Table. In men aged 50–69 years, the age-standardised incidence of early cancer increased by 11% per year (95%CI: +6% to +16%) between 1998 and 2003, corresponding to an absolute mean annual increase of +4.2/100,000 (95%CI: +2.2 to +6.2), and leveled off between 2003 and 2012. The age-standardised incidence of advanced prostate cancer varied between 16.3 and 27.1/100,000 during the 1998 to 2012 time period without noticeable trend. In men aged 70 years and above, no statistically significant trend was observed in the incidence of early or advanced prostate cancer (Table 1, Fig 2 **panel A and B**).

Between 1998 and 2012, 9,397 deaths from prostate cancer occurred (S1 Table). Mortality from prostate cancer decreased steadily and significantly throughout the study period across both age strata (Table 1, Fig 1 **panel A and B**). The decrease was estimated at 3% per year (95%CI: -4% to -1%) in men aged 50–69 years, corresponding to an absolute mean annual change of -0.1/100,000 (95% CI: -0.2 to -0.1) and 2% per year (95%CI: -3% to -1%) in men aged 70 and above, that is, an absolute mean annual change of -0.5/100,000 (95%CI: -0.7 to -0.3) (Table 1).

The annual distribution of the 51,947 cases of prostatectomy registered in Switzerland between 1998 and 2012 and the annual rate of prostatectomy by age group are reported in S3 Table. In men aged 50–69 years, the rate of prostatectomy increased by 40% (95%CI: +20% to +62%) between 1998 and 2002 (absolute mean annual change of +7.6/100,000 (95%CI: +3.6% to +11.5%)), and leveled off afterwards with a maximal peak of 61.3 prostatectomies/100,000 men in 2007. In men aged 70 years and above, the rate of prostatectomy increased less markedly than for those aged 50–69 years (relative and absolute annual increase of 10% and 2.1/100,000, respectively, between 1998 and 2002), and was also stable afterwards. Most of the increase in prostatectomy was ascribed to radical prostatectomy surgery (Table 1). Between 1998 and 2002, the rate of radical prostatectomy increased by 53% (95%CI: +43% to +64%) in men aged 50–69 years (absolute mean annual change of +6.9/100,000 (95%CI: +5.5; +8.2)), and by 54% (95%CI: +23%; +84%) in men aged 70 years and above (absolute mean annual change of +1.3/100,000 (95%CI: +0.6; +2.0)). There was no significant change in trend between 2002 and 2012, and for “other prostatectomies”.

Cases of prostate cancer, deaths from prostate cancer, and prostatectomies were too few before age 50 years to allow statistically meaningful temporal trend analyses (S1 and S3 Tables).

## Discussion

Among men younger than 70 years in Switzerland, we found concomitant increases in prostate cancer incidence and prostatectomy rates between 1998 and 2002/2003, followed by a recent stabilisation until 2012. The incidence trends were mainly driven by early-stage prostate cancer and the surgery trends by radical prostatectomy. Among men aged 70 years or older, the incidence slightly decreased between 1998 and 2012, with a more rapid decrease since 2003. Mortality from prostate cancer steadily decreased in both age groups between 1998 and 2012. These trends altogether may reflect recent changes in screening and clinical workup practices, particularly for men aged 50 to 69 years, with a possible attenuation of overdiagnosis and overtreatment.

Our results are overall consistent with other epidemiological studies. In the US, SEER data indicated that prostate cancer incidence increased for decades up to 1992 before substantially declining from 2011–2012 in men aged 50 years or over, with a large decrease in early-stage prostate cancer [10]. However, the incidence of distant-stage disease has slightly increased in the US since 2010 alongside a levelling off in the mortality decline [10, 21]. These recent results did not concur with our observations for Switzerland.

In Germany, an increase in incidence of prostate cancer up to 2003 was also observed, predominantly for early stage cancers and men aged 60–69 years, with a continuous decrease in mortality between 1999 and 2005 [22]. In the US, incidence and stage-specific trends coincided with the USPSTF recommendation against routine PSA screening, whereas in Switzerland, the stabilisation in trends preceded a 2011 recommendation against routine screening [7]. Changes towards less PSA screening in Switzerland may have occurred before this recommendation. Our finding concurred with a recent regional Swiss study which reported an increase in incidence of prostate cancer in men aged under 65 years in the canton of Zurich up to 2002 and in canton of Ticino up to 2007, followed by a decline [23]. If this trend were to continue in Switzerland, the incidence of early-stage prostate cancer could decrease. Although our analyses were limited to the time period 1998–2012, an increase in the incidence of prostate cancer has been observed since 1983 in Switzerland and attributed to the effect of PSA screening [2].

Others have observed correlations between incidence and surgery rates [24, 25]. Ellison et al. reported a doubling in radical prostatectomy rate in the US between 1989 and 1992 followed by a decrease between 1992 and 1995 [24]. This decrease was most pronounced in older persons, dropping by 51% in men aged 70–74 and by 71% in men aged 75 years and over. In contrast, rates of radical prostatectomy continued to increase in younger men (50–69 years) in the US over the same time period by 8 to 30% depending on the 5 year age group. Increase in treatment rates, particularly radical prostatectomy, in parallel to a rising incidence of prostate cancer during the PSA era with an age and stage migration toward younger age and early stage were also reported in Sweden and in the US [25, 26].

Alternative hypotheses could potentially explain the observed prostate incidence and surgery trends. The increasing incidence could be attributed to the increased use of transurethral resection (TURP) for benign prostate hypertrophy (BPH) from 1970 onwards, where pathology specimens led to discovery of prostate cancer [27]. Furthermore, the greater use of core biopsies and the modification of the Gleason Grading System (2005) could give rise to more diagnoses of prostate cancer. However, as this latter change occurred after the observed leveling off, it is unlikely to explain our results. Both the increased use of TURP and the performance of more core biopsies contribute mainly to an increase in incidence of early prostate cancer, further supporting overdiagnosis, but cannot explain the different trend by age group. The introduction of α-antagonists and 5-α-reductase inhibitors in 1993 as medical treatment for BPH resulted in a decrease in transurethral resections and subsequently histological workup in men over 70 years [28], and could partly explain the discrepant incidence trends between age groups. Improvement over time in prostate cancer staging from additional information provided by novel diagnostic technologies (e.g. ultrasound, bone imaging) might lead to an upward shift in stage distribution. This cannot explain our stage-specific trends as this shift would tend to decrease the incidence of early stage cancers while increasing the incidence of advanced prostate cancers over time.

Better treatment with refined surgical approaches, new modes of radiation, and the introduction of anti-androgen therapy and chemotherapy are the main recognised contributors to the declining prostate cancer mortality [29]. This is supported by the decreasing mortality rate observed in all age groups, even those ages for which prostate cancer screening was less frequent and not recommended. In Switzerland, a steady decline in prostate cancer mortality has been observed since at least 1983 [2], and predates the PSA era. Additionally, a decrease in mortality is also reported in countries without widespread use of PSA screening, such as the UK [1]. Like in Canada [30], but unlike in the US [1], we observed a larger absolute but smaller relative mortality reduction in men older than 70 years.

Our study has several strengths. First, our analyses rely on population-based and high quality data, including cancer registries and nationwide hospital-based statistics. Second, we analysed trends in prostate cancer and surgery during the same time period to compare incidence, mortality, and prostatectomy rates. Third, we used established statistical methods and performed sensitivity analyses about both the allocation of men aged 70–74 years to a 70 and over rather than a 50–74 age group, and our restrictive inclusion criterion for completeness of stage information (60% instead of 90% of cases).

Our study has also limitations, as mentioned in our previous study based on the same type of data and analysis [31]. A sustained increase in incidence confined to early stage tumours, without concomitant increase in mortality of, and a parallel increase in surgery for, prostate cancer can only be suggestive of overdiagnosis and resulting overtreatment, respectively, but no inference can be made. [13, 31, 32]. Although population coverage by Swiss cancer registries is not nationwide, prostate cancer trends do probably not differ substantially across regions, in particular by stage [2]. Thus, the restriction of tumor stage analyses to regions for which staging was nearly systematically documented appears unlikely to alter materially our findings. Surgery coding practices may differ between hospitals and change over time. These are general and well-known issues of studies using medico-administrative data [31, 33]. However, we have taken account of the changes in the coding of prostate surgery over time. Further, as prostate surgeries are major procedures, the probability of cases not being registered and coded is very low. Finally, although systematically recorded, grading information could not be used since a well-known artefactual shift in Gleason score classification from low to high-grade prostate cancer over time prevents any reliable interpretation of temporal trends by grading [19, 34].

In conclusion, the recent leveling off in the incidence of early stage prostate cancer and prostatectomy, along with a stable incidence trend of advanced prostate cancer and a longstanding decrease in prostate cancer mortality for men aged 50 to 69 years suggests that recent favorable changes have occurred in screening and clinical workup practices in Switzerland. The recent stabilisation in incidence of early stage prostate cancers, without any unfavourable impact on trends in advanced prostate cancers and mortality, should contribute to reduce harms from prostate cancer screening. Effective screening strategy for prostate cancer remains to be designed. To reduce overtreatment, active surveillance should be considered for men with low risk prostate cancer [35], and further research on this topic is needed. Meanwhile, careful patients and treatments selection is warranted to preserve the benefits and reduce downstream harms of treatment.

## Supporting information

S1 Table Number of primary invasive cases of prostate cancer, of prostate cancer deaths, and age-standardised (European population) incidence and mortality rates of prostate cancer per 100,000 inhabitants by year and age group in Switzerland, 1998–2012. (DOCX)

S2 Table Age-standardised (European population) incidence rates of prostate cancer per 100,000 inhabitants, by year and stage in Switzerland, 1998–2012. (DOCX)

S3 Table Annual numbers and age-standardised (European population) rate of prostatectomy per 100,000 inhabitants and age group in Switzerland, 1998–2012. (DOCX)
