# Supplementary material for: Recent incidence and surgery trends for prostate cancer: Towards an attenuation of overdiagnosis and overtreatment?
Source: PLoS One. 2019 Feb 4;14(2):e0210434. doi: 10.1371/journal.pone.0210434 (PMC6361620; doi:10.1371/journal.pone.0210434)
Supplement: S1 Table — (DOCX) [file pone.0210434.s001.docx]

**Supporting Information**

**Table S1.**

|  | **all ages** | | **age 0-49** | | **age 50-69** | | **age ≥ 70** | |
| --- | --- | --- | --- | --- | --- | --- | --- | --- |
| **year** | **incidence (cases)** | **mortality (cases)** | **incidence (cases)** | **mortality (cases)** | **incidence (cases)** | **mortality (cases)** | **incidence (cases)** | **mortality (cases)** |
| 1998 | 119.4 (2693) | 28.7 (552) | 0.7 (13) | 0.0 (0) | 53.7 (1083) | 4.7 (78) | 65.1 (1597) | 23.8 (474) |
| 1999 | 119.0 (2730) | 32.1 (642) | 0.5 (10) | 0.0 (1) | 53.5 (1100) | 5.4 (91) | 65.0 (1620) | 27.3 (550) |
| 2000 | 122.5 (2836) | 29.7 (602) | 0.8 (17) | 0.0 (0) | 57.4 (1198) | 5.0 (85) | 64.3 (1621) | 25.2 (517) |
| 2001 | 133.6 (3136) | 28.3 (596) | 1.0 (22) | 0.0 (0) | 67.9 (1436) | 4.9 (86) | 64.6 (1678) | 23.8 (510) |
| 2002 | 136.6 (3257) | 25.4 (533) | 0.6 (12) | 0.0 (0) | 72.6 (1565) | 3.9 (69) | 63.4 (1680) | 21.1 (464) |
| 2003 | 133.7 (3238) | 28.1 (608) | 1.3 (29) | 0.0 (1) | 72.2 (1589) | 4.6 (83) | 60.2 (1620) | 23.4 (524) |
| 2004 | 143.3 (3540 | 27.8 (613) | 1.1 (25) | 0.0 (0) | 78.2 (1755) | 5.1 (95) | 64.0 (1760) | 22.5 (518) |
| 2005 | 132.9 (3425) | 25.3 (586) | 1.3 (30) | 0.0 (0) | 70.4 (1641) | 3.6 (68) | 61.2 (1754) | 21.6 (518) |
| 2006 | 130.8 (3609) | 24.8 (595) | 1.4 (37) | 0.1 (2) | 71.4 (1786) | 3.7 (77) | 58.0 (1786) | 19.8 (516) |
| 2007 | 135.7 (3810) | 23.5 (585) | 1.9 (50) | 0.0 (0) | 76.0 (1951) | 3.7 (78) | 57.7 (1809) | 18.9 (507) |
| 2008 | 133.4 (3847) | 24.6 (621) | 1.4 (38) | 0.0 (1) | 73.4 (1933) | 3.4 (73) | 58.5 (1876) | 20.0 (547) |
| 2009 | 134.8 (3958) | 21.7 (588) | 1.2 (33) | 0.2 (3) | 75.9 (2049) | 2.8 (62) | 57.7 (1876) | 18.3 (523) |
| 2010 | 124.4 (4012) | 23.7 (734) | 1.6 (51) | 0.0 (1) | 72.2 (2144) | 3.4 (85) | 50.5 (1817) | 20.1 (648) |
| 2011 | 130.8 (4050) | 24.2 (792) | 1.2 (37) | 0.1 (2) | 75.6 (2164) | 3.4 (91) | 54.0 (1849) | 20.6 (699) |
| 2012 | 115.5 (3845) | 20.8 (750) | 1.3 (42) | 0.1 (1) | 67.0 (2054) | 3.6 (103) | 47.2 (1749) | 17.4 (646) |
| 1998-2012 | 129.6 (51986) | 25.4 (9397) | 1.2 (446) | 0.0 (12) | 69.4 (25448) | 4.0 (1224) | 59.0 (26092) | 21.3 (8161) |

Data source: National Institute of Cancer Epidemiology and Registration (NICER) and Federal Statistical Office (FSO)
