# Supplementary material for: Recent incidence and surgery trends for prostate cancer: Towards an attenuation of overdiagnosis and overtreatment?
Source: PLoS One. 2019 Feb 4;14(2):e0210434. doi: 10.1371/journal.pone.0210434 (PMC6361620; doi:10.1371/journal.pone.0210434)
Supplement: S2 Table — (DOCX) [file pone.0210434.s002.docx]

**Supporting Information**

**Table S2.**

|  | **all ages** | | | **age 0-49** | | | **age 50-69** | | | **age ≥ 70** | | |
| --- | --- | --- | --- | --- | --- | --- | --- | --- | --- | --- | --- | --- |
| **year** | **early** | **advanced** | **unknown** | **early** | **advanced** | **unknown** | **early** | **advanced** | **unknown** | **early** | **advanced** | **unknown** |
| 1998 | 59.1 | 34.0 | 10.8 | 0.3 | 0.0 | 0.0 | 33.1 | 19.1 | 1.9 | 22.9 | 11.2 | 5.5 |
| 1999 | 67.8 | 49.3 | 9.3 | 0.3 | 0.3 | 0.0 | 37.8 | 23.2 | 1.5 | 26.7 | 20.4 | 4.5 |
| 2000 | 55.7 | 42.4 | 11.4 | 0.3 | 0.3 | 0.0 | 36.2 | 21.5 | 1.2 | 16.2 | 16.9 | 5.4 |
| 2001 | 84.2 | 51.5 | 13.8 | 1.2 | 0.3 | 0.0 | 49.0 | 27.1 | 2.6 | 29.8 | 19.6 | 6.7 |
| 2002 | 79.7 | 45.6 | 12.1 | 0.5 | 0.0 | 0.0 | 53.3 | 23.6 | 2.6 | 23.3 | 18.1 | 5.1 |
| 2003 | 81.2 | 38.1 | 10.4 | 1.1 | 0.6 | 0.0 | 50.9 | 16.3 | 1.4 | 26.4 | 17.9 | 3.0 |
| 2004 | 95.0 | 42.5 | 11.1 | 1.3 | 0.8 | 0.0 | 61.7 | 24.0 | 1.6 | 29.0 | 14.2 | 6.1 |
| 2005 | 77.6 | 40.0 | 10.5 | 0.3 | 0.5 | 0.0 | 43.5 | 19.3 | 1.4 | 31.0 | 16.3 | 6.2 |
| 2006 | 80.9 | 37.8 | 10.5 | 0.8 | 0.8 | 0.0 | 53.0 | 20.4 | 2.6 | 25.9 | 13.9 | 5.6 |
| 2007 | 76.7 | 38.0 | 9.9 | 0.9 | 1.1 | 0.0 | 46.8 | 22.0 | 1.6 | 26.4 | 11.9 | 5.6 |
| 2008 | 82.7 | 34.7 | 11.6 | 0.7 | 0.2 | 0.0 | 51.4 | 18.1 | 3.8 | 27.4 | 12.9 | 5.1 |
| 2009 | 85.3 | 37.7 | 15.8 | 1.4 | 0.0 | 0.3 | 53.8 | 21.1 | 4.0 | 27.6 | 14.2 | 8.4 |
| 2010 | 83.9 | 35.2 | 14.8 | 2.5 | 0.8 | 0.0 | 53.9 | 16.7 | 4.4 | 25.0 | 15.0 | 7.2 |
| 2011 | 97.2 | 37.8 | 9.6 | 1.6 | 0.3 | 0.2 | 62.8 | 21.4 | 2.8 | 30.4 | 13.6 | 4.7 |
| 2012 | 82.9 | 36.7 | 13.1 | 1.0 | 0.8 | 0.2 | 51.6 | 19.9 | 5.7 | 28.7 | 13.3 | 5.1 |

Data source: National Institute of Cancer Epidemiology and Registration (NICER) limited to 3 regions (cf. methods section)
