# Supplementary material for: Recent incidence and surgery trends for prostate cancer: Towards an attenuation of overdiagnosis and overtreatment?
Source: PLoS One. 2019 Feb 4;14(2):e0210434. doi: 10.1371/journal.pone.0210434 (PMC6361620; doi:10.1371/journal.pone.0210434)
Supplement: S3 Table — (DOCX) [file pone.0210434.s003.docx]

**Supporting Information**

**Table S3.**

|  | **all ages** | **age 0-49** | **age 50-69** | **age ≥ 70** |
| --- | --- | --- | --- | --- |
| **year** | **rate (cases)** | **rate (cases)** | **rate (cases)** | **rate (cases)** |
| 1998 | 40.3 (1541) | 0.2 (8) | 19.7 (673) | 20.3 (860) |
| 1999 | 53.1 (2042) | 0.2 (6) | 28.4 (986) | 24.6 (1050) |
| 2000 | 57.4 (2228) | 0.5 (17) | 31.8 (1124) | 25.2 (1087) |
| 2001 | 71.7 (2821) | 0.5 (19) | 42.8 (1535) | 28.3 (1267) |
| 2002 | 76.8 (3063) | 0.7 (28) | 47.5 (1738) | 28.6 (1297) |
| 2003 | 80.5 (3281) | 1.0 (39) | 50.1 (1879) | 29.4 (1363) |
| 2004 | 80.2 (3347) | 0.6 (23) | 49.6 (1900) | 30.1 (1424) |
| 2005 | 87.1 (3702) | 1.0 (39) | 54.0 (2117) | 32.2 (1546) |
| 2006 | 84.5 (3691) | 0.8 (35) | 51.3 (2058) | 32.4 (1598) |
| 2007 | 96.2 (4274) | 1.4 (58) | 61.3 (2525) | 33.6 (1691) |
| 2008 | 93.9 (4281) | 1.0 (43) | 59.6 (2524) | 33.3 (1714) |
| 2009 | 92.9 (4323) | 1.2 (55) | 59.8 (2604) | 31.9 (1664) |
| 2010 | 90.4 (4334) | 0.8 (39) | 56.7 (2544) | 32.9 (1751) |
| 2011 | 94.5 (4594) | 1.1 (51) | 60.7 (2772) | 32.7 (1771) |
| 2012 | 88.2 (4425) | 1.1 (53) | 54.0 (2511) | 33.0 (1861) |

Data source: Federal Statistical Office (FSO), Swiss Hospital Medical Statistics.
